# Editorial: Performance and Participation Outcomes for Individuals With Neurological Conditions

**DOI:** 10.3389/fneur.2020.00878

**Published:** 2020-09-15

**Authors:** Naomi Josman, Lisa Tabor Connor, David J. Lin

**Affiliations:** ^1^Department of Occupational Therapy, Faculty of Social Welfare & Health Science, University of Haifa, Haifa, Israel; ^2^Program in Occupational Therapy, Departments of Neurology & Social Work, Washington University School of Medicine, St. Louis, MO, United States; ^3^Department of Neurology, Center for Neurotechnology and Neurorecovery, Massachusetts General Hospital and Harvard Medical School, Boston, MA, United States; ^4^Division of Neurocritical Care and Emergency Neurology, Department of Neurology, Massachusetts General Hospital, Boston, MA, United States

**Keywords:** central nervous system deficits, neurorehabiliation, cognition, neurorecovery, neuroplasticity, stroke recovery

People suffering from neurological conditions such as Parkinson's disease, stroke, and brain injury encounter and endure numerous situations in their daily lives that cause excess disability and restrict full participation in their meaningful activities.

Neuroscience and rehabilitation science are complementary disciplines, engaged in the exploration of the human Central Nervous System (CNS) and the amelioration of functional disability. There is often, however, a lack of discourse between these disciplines, which precludes opportunities for meaningful exchange and synergistic collaboration to improve the lives of people who suffer from a disability.

The goal of this current theme issue was to foster original research papers in neuroscience and rehabilitation science that may serve to address the existing disconnect between disciplines. This platform offered an opportunity for a meaningful exchange of ideas, findings, and practices toward ultimately promoting knowledge, improving clinical practice, and reducing performance deficits and participation restrictions for people with neurological ailments and diseases. This broader, biopsychosocial approach to understanding rehabilitation and recovery from neurological deficits, embraces the concept that true understanding of recovery and living with a chronic condition, goes beyond knowledge of disease mechanisms. Social determinants and secondary disease sequelae may have a significant impact on functional performance and participation.

The initial call for papers invited researchers and practitioners to submit research papers on the topic of “Performance and Participation Outcomes for Individuals with Neurological Conditions.” This focused topic recognized the need for a more comprehensive and integrative perspective in research and practice to address this disconnect between neuroscience and rehabilitation. This perspective should thus reveal neglected key domains, as well as incorporate a range of components intricately involved in human performance, such as motor, sensory, cognitive, and emotional components. Such integration may serve to significantly enhance the functional outcomes for persons suffering from any of the range of neurological conditions.

The theme call aimed at addressing these respective issues by soliciting original research papers, review articles, or meta-analyses, covering topics ranging from basic research to translational studies anchored in neuroscience or rehabilitation.

The encouraging response to our call yielded 22 submitted papers that underwent a peer-review process. Ultimately, 16 papers were adjudged to favorably meet the theme call objectives and scientific standards. As this special issue was designed to organize our thinking around outcome, rather than disease, we provide a classification of the articles by their primary outcome focus. We categorized the papers as addressing prognostic indicators of recovery (4 articles), performance (4 articles), or participation (8 articles). Schliep et al.; Valè et al.; Wang, Wang et al.; and Sul et al. addressed the need for greater understanding of prognostic indicators of recovery such as lesion location, behavioral characteristics, EMG assessment in motor disorders, and the initiation of swallowing in disorders of consciousness. Performance outcomes and their contributors was the focus of Fasoli and Adans-Dester, Ranford et al., Yael et al., and McCambridge et al.. The vast majority of the papers included in this special issue cluster around the multifactorial outcome of participation: Rotenberg et al., Malone et al., Yosef et al., Wang, Chan et al., Toglia et al., Erler et al., Nicholas et al., and Cattaneo et al..

The breadth of neurological conditions and methodological approaches in this issue is astounding. Although broad in scope, our focus on the larger outcomes of people with neurological conditions, we believe, enables us to glean principles for maximizing performance and participation at the highest conceptual level. One of the most striking themes that emerged from this collection, for example, is that cognitive and emotional factors are of utmost importance for the performance of motor-intensive activities and participation in life activities that are motorically-demanding in diseases classically thought of as diseases of the motor system. Moreover, social support is a major predictor of resumption of pre-disease activities.

Of course, putting the focus on outcome introduces factors that are outside the purview of neurological science and will not replace studies focused on disease mechanisms that foster new treatment approaches aimed at treating or curing disease. The vast majority of the patients living with these neurological conditions will be doing just that—living with their chronic conditions. Our hope is that a more holistic scientific treatment of factors predicting outcome will advance our rehabilitation attempts to promote better living for people with neurological conditions.

## Author Contributions

All authors listed have made a substantial, direct and intellectual contribution to the work, and approved it for publication.

## Conflict of Interest

The authors declare that the research was conducted in the absence of any commercial or financial relationships that could be construed as a potential conflict of interest.

